# Black Carbon: The Dark Horse of Climate Change Drivers

**DOI:** 10.1289/ehp.119-a172

**Published:** 2011-04

**Authors:** Charles W. Schmidt

**Affiliations:** **Charles W. Schmidt**, MS, an award-winning science writer from Portland, ME, has written for *Discover Magazine*, *Science*, and *Nature Medicine*

For decades, efforts to slow global warming have mostly aimed to limit heat-trapping emissions of carbon dioxide (CO_2_). Now scientists are pointing to a different class of warming agents they say also must be targeted to keep global temperatures in check. Dubbed “short-lived climate forcings” (SLCFs), these other emissions—namely, black carbon particles, methane, hydrofluorocarbons, and tropospheric ozone—are even more powerful than CO_2_ in terms of their warming potential. But they persist in the atmosphere for much shorter durations than CO_2_, which can linger airborne for hundreds to thousands of years.^1^

Steve Seidel, vice president for policy analysis at the Pew Center on Global Climate Change, says the recent emphasis on SLCFs represents new policy thinking on climate change. “We thought the Kyoto Protocol and its follow-on agreements would get us to where we need to be, but that’s not working out the way we hoped it would,” he says. “So, we’re broadening the discussion and opening up new pathways for going forward.”

Given the enormity of human emissions, many climate scientists believe CO_2_ will one day become the dominant force behind climate change. But for now, CO_2_ and the SLCFs are nearly on par in terms of their climate changing effects, according to Veerabhadran Ramanathan, a professor at The Scripps Institute of Oceanography.

In a report published in February 2011, the United Nations Environment Programme (UNEP) called attention to SLCFs, claiming their emissions must be cut together with CO_2_ in order to prevent global temperatures from crossing a dangerous threshold.^2^ Doing that would offer health benefits too, UNEP stated, because SLFCs are also toxic air pollutants. Particulate emissions from diesel exhaust—a major source of black carbon—have been linked to lung and heart disease as well as cancer.^3^ But where it would take a transformation of the energy sector (at a cost of trillions of dollars over multiple decades^1^) to drop CO_2_ emissions enough to influence the climate, cutting SLCFs to achieve a similar goal could be achieved with current technologies under policy frameworks that are already in place, such as clean air regulations, according to Seidel.

## Dark and Dirty

Among the SLCFs, black carbon garners the most attention because its climate and health effects are greater than those of the others, says Mark Jacobson, a professor in the Stanford University Department of Energy Resources Engineering. Evidence on black carbon’s climate impacts has been building since at least the mid-1990s, when Ramanathan and colleague Paul Cruzan, a Nobel prize–winning atmospheric chemist from the Max Planck Institute for Chemistry, first speculated that “brown clouds” laden with the dark particles influence weather patterns over South Asia, a hypothesis that was supported by future research.^4^

But the way black carbon affects the climate is nuanced and hard to study, and it’s only recently that the science has begun to mature to the degree that policies to limit emissions can be proposed on climatic grounds, says Drew Shindell, a scientist with the Goddard Institute for Space Studies at the National Aeronautics and Space Administration (NASA), who led the panel that produced the new report by UNEP. “What we’re seeing now with the UNEP document and other more recent papers are attempts to generate the first cohesive picture of black carbon’s effects on the climate and ways to address it,” Seidel says.

Spewed into the air by diesel engines, dirty cookstoves, and open burning, black carbon is the material that burns in an orange flame, explains Tami Bond, an affiliate professor of atmospheric sciences at the University of Illinois at Urbana–Champaign. “What you see in fire is black carbon glowing,” she says. What escapes to the air from fire, Bond adds, are agglomerated particles of nearly pure carbon, each several thousand times smaller than the width of a human hair.

Those particles absorb sunlight in all its wavelengths and transfer its warmth to the atmosphere. With roughly a million times the heat-trapping power of CO_2_,^5^ black carbon can travel long distances on air currents. If it falls out with precipitation on snowpack or ice, it absorbs heat and accelerates melting by interfering with how those white surfaces reflect sunlight back to space.^6^

But black carbon is also co-emitted with other particles that reflect more sunlight than they absorb. And these other specks of ash and organic materials have a net cooling effect, such that combustion emissions will warm the air only as much as their black carbon content allows. With a roughly 1:1 ratio of organic^7^ to black carbon particles, diesel emissions top the list in terms of their climate warming potential, according to Jacobson.

Emissions from solid fuel combustion—namely, from cookstoves that burn animal dung, wood, and other types of biomass—follow with a ratio of organic to black carbon particles of 4:1. Open fires tend to smolder and eject a lot of ash particles that reflect sunlight, but even so, they exert a net warming effect on the atmosphere, Jacobson says. On the other hand, emissions from forest fires, with an 8:1 organic to black carbon particle ratio, cool the atmosphere in the short run but lead to warming later because of the massive amounts of CO_2_ they put into the air, he says.

## Climatologic Impacts

About 77% of the estimated 8,000 kilotons of black carbon emitted globally every year come from the developing world, discharged mainly from cookstoves, open burning, and old diesel engines,^8^ which means the focus of cleanup lies largely with poorer countries, possibly with the financial and technical support from developed countries, according to Seidel. Wealthier nations such as the United States, on the other hand, emit much less black carbon, and diesel engines account for the vast majority of those emissions.^8^

North Amerian emissions dominate when it comes to the black carbon falling on ice in Greenland, Shindell says, while European emissions dominate what reaches the rest of the Arctic. “The largest black carbon source in both North America and Europe is diesel, so I think it’s safe to say that’s the biggest [contributor from these countries],” he says.

As for additional contributions from northern industrialized countries—and Arctic ice sheets are known to be most vulnerable to black carbon emissions from locales north of the 40th parallel^8^—Shindell also cites forest fires and residential woodstoves and fireplaces. But he emphasizes that the role of black carbon in Arctic melting isn’t fully understood and that much of the ice losses there so far probably result from greenhouse gases.^9^

“What we can say is that black carbon from northern countries is the dominant contributor to darkening of Arctic snow, which is at least partly responsible for melting,” he says. “It’s hard to be more definitive as black carbon trends during the last few decades, when melting has accelerated greatly, seem not to be large—roughly flat, really—but we only have data for the Western Hemisphere, and even that is fairly sparse.”

Unlike greenhouse gases, which float around the planet on long time scales, black carbon travels in the air for only a week or 10 days before it washes out of the atmosphere.^2^ Its effects are therefore more regional than global, and its influence on the climate results from both its radiative heating effects and its ability to disrupt cloud formation and rainfall.^5^

Daniel Rosenfeld, a professor of atmospheric sciences at the Hebrew University of Jerusalem, says much about black carbon’s influence on weather remains unknown, however. Ordinarily, airborne particulates seed clouds, he explains, but black carbon particles can get hot enough to vaporize water and prevent clouds from forming at all. Cloud losses result in more heating of the ground, Jacobson adds. And that reduces air pressure over land, which draws air currents from areas of higher pressure, resulting in higher windspeeds.

But depending on a range of conditions, including the particulate makeup of the pollution and topographical features of the land, particle emissions can also seed clouds made up of unusually small droplets. These clouds don’t coalesce into denser forms that would otherwise fall as rain, Rosenfeld explains. The result is more clouds but less rain than usual, with commensurate impacts on water supplies and agriculture.^10^

The implications of these impacts are a focus of intense research, but in the meantime, Erika Rosenthal, a staff attorney at Earth Justice, says that South Asian monsoons now come roughly two to three weeks earlier than usual, perhaps because of the region’s heavily polluted air.^11^ “And that’s crucial for farmers who feed a quarter of the world’s population,” she says.

Still, Rosenfeld cautions that the science in this area is an evolving story. “It’s very difficult for the scientific community to tease out these effects,” he says. “We’re trying to distinguish radiative effects from how particles absorb solar rays apart from air pollution’s effects on clouds, precipitation, and evaporative forces. This is a very big challenge in the field.”

## Policy Implications

Just how climate-related concerns about black carbon will drive policy remains to be seen. Policy momentum on SLCFs is picking up on certain fronts. UNEP’s 2011 report presents 16 strategies to stanch the flow of SLCFs into the atmosphere, among them capping fugitive methane emissions from industry and agriculture, banning open-field burning of agricultural waste, taking old diesel vehicles off the road, and substituting traditional biomass cookstoves in the developing world with cleaner models. If achieved within the next 20 years, those measures could halve the rate of climate change expected by mid-century while avoiding some 0.7–4.6 million premature deaths that would have resulted from poor air quality, UNEP asserts.^2^

Meanwhile, a task force convened by the Arctic Council, an intergovernmental forum of circumpolar nations, is investigating ways to lower SLCF emissions with an eye toward limiting rates of ice sheet melting in the near term.^12^ The measures will be identified in a report to be presented at the council’s next ministerial meeting, in Nuuk, Greenland, on 12 May 2011. Finally, the U.S. Environmental Protection Agency (EPA) is set to release a report to Congress in April 2011 detailing sources of black carbon and cost-effective ways to minimize its health and climate impacts. EPA officials declined to comment on the report in advance of publication.

According to Seidel, the United Nations Framework Convention on Climate Change isn’t well suited for negotiations on black carbon; “You’re more likely to see this move forward under regional frameworks focused on air quality,” he says. As an example, he cites the Montréal Protocol, which successfully phased out the chlorofluorocarbons that degrade the ozone layer.

In the United States, black carbon reductions apply mainly to diesel standards, which have already been tightening since the 1970s in response to health needs. The California Air Resources Board (CARB) led the charge, issuing the first statewide regulation on diesel emissions from heavy trucks in the late 1980s. Since then, regulations have steadily tightened in California,^13^ and the U.S. EPA has followed suit.^14^

In 2006 the EPA adopted an ultra-low-sulfur diesel requirement for on-road vehicles that dropped allowable concentrations from 500 to 15 ppm, and the agency is now expanding that rule to cover more transportation sources, including off-road vehicles, railroads, and ships. Ultra-low-sulfur diesel fuels end up reducing black carbon emissions because they allow for the use of particulate exhaust filters, which would have been “poisoned” (rendered ineffectual) by sulfates. Since 2007, the EPA has mandated that all new on-road vehicles be equipped with advanced emission controls that require the new cleaner diesel fuels to run properly.

Ramanathan’s group recently published a study showing that California’s black carbon emissions dropped 50% over the period 1989–2008.^15^ (That’s according to measurements collected at 22 sites through California’s Interagency Monitoring of Protected Visual Environments program.) The study results also suggested those reductions were accompanied by a corresponding 50% drop in black carbon’s warming effect (or more specifically, its “radiative forcing”) over the whole state of California.

But Bart Croes, chief of the CARB Research Division, says there’s no plan to tighten the state’s diesel regulations further in response to climate concerns. “Public health is the major driver behind these regulations, and they appear to have also reduced climate impacts,” he says. “So we see no need to modify our regulations [specifically] to address the climate. What we’re doing for public health is also exactly what we should be doing for the climate.”

California now mandates retrofits to bring all pre-2007 on-road diesel truck and buses in line with current particle emissions regulations. According to CARB calculations, these older vehicles accounted for 95% of all diesel particulate emitted from on-road trucks and buses in California in 2010. The estimated cost to retrofit trucks and buses in the state will be $2.2 billion from 2012 to 2025.^16^ Of course, estimated costs nationwide are far higher: a 2009 report on black carbon published by the Pew Center on Global Climate Change cited data showing it would cost $32 billion to retrofit 54% of the estimated 5.4 million heavy-duty on-road diesel vehicles in the United States.^5^

That’s a lot of money. But considering that 90% of U.S. black carbon emissions come from the transportation sector, mainly diesel vehicles, it’s also just part of what the nation would have to pay in order to meet UNEP’s aim to install diesel particle filters for on- and off-road vehicles and to eliminate high-emitting on- and off-road vehicles, which are 2 of the 16 strategies identified in its report.^2^

Meanwhile, looking for budget-slashing opportunities, President Obama recently cut 2012 funding for the Diesel Emissions Reduction Program, which gives EPA grant and loan authority to fund the retrofitting or replacement of existing diesel vehicles. The alternative, of course, is to refrain from mandatory retrofitting and take the vehicles off the road through attrition.

But that leads to an intriguing question: If—as is the case in California—the United States is unwilling or unlikely to impose further tightening of diesel regulations in response to climate concerns, how does the emerging evidence on black carbon influence environmental policy here? Seidel says there is no evidence that cleaning up diesels in the United States will have the biggest, let alone the most cost-effective, impacts on slowing warming in the Arctic. Yet Rosenthal argues that U.S. contributions to Arctic black carbon pollution constitute an imperative for the country to clean up its diesel emissions faster.

But most of the opportunity to reduce emissions are found in the developing world, she adds, where diesel standards aren’t as stringent, and where cookstoves and open burning pose major environmental problems. “The science and policy dilemmas are complicated,” Rosenthal says. “But we need to make decisions about this now.”

## Figures and Tables

**Figure:**
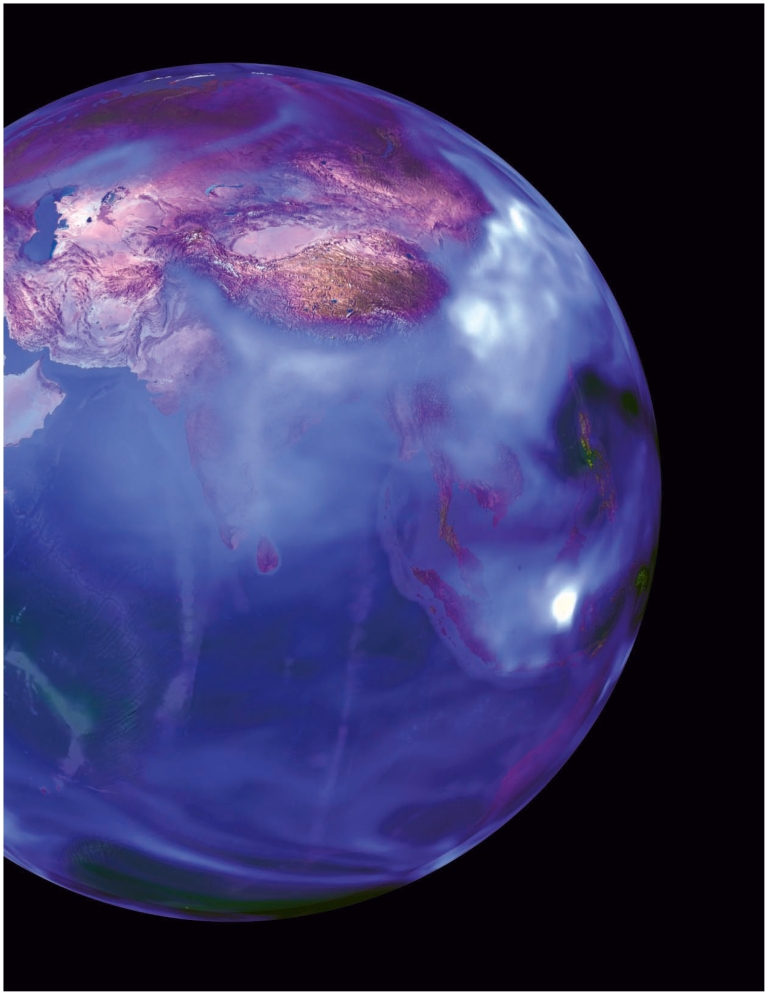
More than three-quarters of the world’s black carbon is thought to come from developing countries, discharged from cookstoves, open burning, and older diesel engines. This data visualization uses data from NASA’s GEOS-5 Goddard Chemistry Aerosol and Transport (GOCART) climate model to show atmospheric concentrations of black carbon on 26 September 2009. Aerosol optical thickness ranges nonlinearly from 0.002 (transparent) to 0.02 (purple) to 0.2 (white). Animations of global black soot transport are available at http://tinyurl.com/64nbykb and http://tinyurl.com/69w9s6z.

## References

[1] Ramanathan V, Victor DG (2010). To Fight Climate Change, Clear the Air. New York Times, Opinion section, online edition.

[2] UNEP (2011). Integrated Assessment of Black Carbon and Tropospheric Ozone: Summary for Decision Makers.

[3] OEHHA (2007). Air Toxicology and Epidemiology. Health Effects of Diesel Exhaust: A Fact Sheet by Cal/EPA’s Office of Environmental Health Hazard Assessment and the American Lung Association.

[4] Ramanathan V (2002). The Indian Ocean experiment and the Asian brown cloud. Curr Sci.

[5] Bachmann J (2009). Black Carbon: A Science/Policy Primer.

[6] Doherty SJ (2009). Black Carbon in Arctic Snow and Its Effect on Surface Albedo. American Geophysical Union, Fall Meeting, 2009 Abstract #A34B-05.

[b7-ehp-119-a172] 7“Organic carbon” is a term of art referring to organic compounds that contain carbon. Organic carbon is not as black as black carbon, and it absorbs solar heat much less effectively.

[8] Bice K (2009). Black Carbon: A Review and Policy Recommendations.

[9] Schmidt CW (2011). Out of equilibrium? The world’s changing ice cover. Environ Health Perspect.

[10] Jacobson MZ (2010). Short-term effects of controlling fossil-fuel soot, biofuel soot and gases, and methane on climate, Arctic ice, and air pollution health. J Geophys Res.

[11] Ramanathan V (2008). Atmospheric Brown Clouds: Regional Assessment Report. Summary.

[12] Arctic Council Task Force on SLF Meeting [website].

[13] California Diesel Fuel Program [website] http://tinyurl.com/67crxbd.

[14] Fuels and Fuel Additives [website].

[15] Bahadur R (2011). Impact of California’s air pollution laws on black carbon and their implications for direct radiative forcing. Atmos Environ.

[16] CARB Truck and Bus 2010 [website]. Appendix I: Costs and Cost Methodology.

